# *In Silico* Structural Characterization and Hypoglycemic Potential of a Novel Fucose-Specific Lectin (MEP5) from *Morchella esculenta*

**DOI:** 10.3390/foods15091493

**Published:** 2026-04-24

**Authors:** Wanchao Chen, Peng Liu, Wen Li, Di Wu, Zhong Zhang, Yan Yang

**Affiliations:** Institute of Edible Fungi, Shanghai Academy of Agricultural Sciences, National Engineering Research Center of Edible Fungi, Key Laboratory of Edible Fungi Resources and Utilization (South), Ministry of Agriculture and Rural Affairs, Shanghai 201403, China; liupeng@saas.sh.cn (P.L.); liwen@saas.sh.cn (W.L.); wudi@saas.sh.cn (D.W.); zhangzhong@saas.sh.cn (Z.Z.)

**Keywords:** *Morchella esculenta*, fucose-specific lectin, *α*-glucosidase, DPP-IV inhibitory peptides, simulated gastrointestinal digestion, molecular docking

## Abstract

Natural food-derived proteins are increasingly explored as alternatives to synthetic inhibitors for managing Type 2 diabetes mellitus. Despite the recognized health-promoting properties of *Morchella esculenta*, the potential of its bioactive proteins to modulate glucose metabolism remains largely unexplored. This study systematically investigated the structural basis and hypoglycemic mechanisms of MEP5 (*Morchella esculenta* Protein 5), a fucose-specific lectin from *M. esculenta*, using an integrated *in silico* pipeline. MEP5 (33.12 kDa) adopts a stable *β*-sheet-rich conformation and harbors a conserved fucose-binding carbohydrate-recognition domain. Protein–protein docking revealed that intact MEP5 binds directly to surface glycans of human *α*-glucosidase, generating steric hindrance that obstructs the catalytic pocket. Simulated gastrointestinal digestion yielded a highly bioavailable peptide profile. Following a rigorous multiparametric screening for toxicity, allergenicity, and water solubility, 11 short oligopeptides were identified as potent dipeptidyl peptidase-IV (DPP-IV) inhibitors. Molecular docking demonstrated that the top-ranked peptides, QPPR, DGTY, and DPDSH, occupy the S2 pocket of DPP-IV and form hydrogen bonds with catalytic triad residues (Ser630/His740). These findings delineate a dual-stage hypoglycemic mechanism, pre-digestion enzymatic blockade and post-digestion incretin regulation, and support the potential of MEP5 as a multifunctional candidate for glucose homeostasis-oriented functional foods.

## 1. Introduction

Type 2 diabetes mellitus (T2DM) has emerged as a critical global health challenge, characterized by chronic hyperglycemia and metabolic dysfunction [1]. A primary therapeutic strategy for managing T2DM involves the inhibition of key enzymes involved in carbohydrate digestion, such as *α*-glucosidase and *α*-amylase, as well as the modulation of dipeptidyl peptidase-IV (DPP-IV) to enhance insulin secretion [2]. While synthetic inhibitors like acarbose are effective, they are often associated with gastrointestinal side effects, prompting increased interest in identifying natural, food-derived bioactive compounds with hypoglycemic potential [3,4]. Nevertheless, it is important to recognize that “natural” does not automatically equate to “safe”; food-derived bioactive proteins and peptides must undergo rigorous evaluation for potential toxicity, allergenicity, and undesirable interactions before they can be considered for functional food applications. In this context, computational approaches offer a valuable first-tier screening strategy to prioritize candidates with favorable safety and efficacy profiles.

Edible mushrooms have long been recognized as a reservoir of functional ingredients, including polysaccharides, phenolics, and bioactive proteins [5]. Among these, *Morchella esculenta* (Morel), a highly prized ascomycete fungus, is valued not only for its unique flavor but also for its diverse pharmacological properties, such as antioxidant, anti-inflammatory, and immunomodulatory activities [6,7]. While much research has focused on morel polysaccharides [8], the bioactive proteins [9], particularly lectins, have remained underexplored despite their significant potential in metabolic regulation.

Lectins constitute a unique class of carbohydrate-binding proteins ubiquitously distributed across edible fungi. They possess the inherent capacity to specifically recognize and reversibly bind to glycoconjugates on cell surfaces or enzyme structures, thereby modulating diverse biological processes, including glucose metabolism [10]. To contextualize the present investigation, it is instructive to consider the broader landscape of mushroom lectins in glycemic control. Among the most extensively studied mushroom lectins is ABL from *Agaricus bisporus* (white button mushroom), which has demonstrated multifaceted antidiabetic properties. Early studies by Ewart et al. first revealed that ABL stimulates insulin and glucagon release from isolated rat pancreatic islets in a glucose-independent manner [11]. Subsequent investigations by Ahmad et al. confirmed that the PHA-B fraction of *A. bisporus* lectin dose-dependently enhances insulin secretion and promotes Ca^2+^ uptake in islets of Langerhans [12]. More recently, Wang et al. demonstrated that ABL administration could partially reverse impaired *β*-cell growth potential by regulating key cell cycle proteins, suggesting therapeutic potential in preventing and/or treating diabetes [13]. Similarly, lectins from *Pleurotus ostreatus* (POL) have been structurally characterized as homodimeric proteins with calcium-dependent carbohydrate-binding sites, and crude extracts of *P. ostreatus* have shown hypoglycemic effects in streptozotocin-induced diabetic rats [14,15]. Notably, Téllez-Téllez et al. comprehensively reviewed the antidiabetic activities of mushroom-derived bioactive compounds and highlighted that lectins, alongside polysaccharides and other fungal proteins, represent an underexplored reservoir of *α*-glucosidase inhibitors and insulin secretagogues [16]. Despite these promising precedents in related fungal taxa, the hypoglycemic potential of lectins from *Morchella* species remains largely uncharacterized, representing a critical gap in the current knowledge base.

Recently, our group identified and characterized a novel fucose-specific lectin from *M. esculenta*, designated as MEP5 (*Morchella esculenta* Protein 5) [17]. Our previous study demonstrated that MEP5 is a 33.12 kDa protein that exerts potent hepatoprotective effects. It significantly ameliorated non-alcoholic fatty liver disease (NAFLD) in high-fat-diet-induced mice by normalizing lipid profiles and modulating the gut–liver axis via the MAPK signaling pathway [17,18]. Given the intimate crosstalk between lipid and glucose metabolism, often referred to as “glycolipid metabolism”, and the observed ability of MEP5 to regulate key metabolic genes such as G6pc1 and PPAR*α*, it is highly probable that MEP5 also possesses hypoglycemic properties [17].

Beyond their potential direct action on digestive enzymes via carbohydrate recognition [10], food proteins can undergo hydrolysis during gastrointestinal transit to release bioactive peptides. These peptides often exhibit superior bioactivity and bioavailability compared to their parent proteins [19]. In this context, *in silico* approaches, including molecular docking and simulated gastrointestinal digestion, have become a robust and efficient approach in food science to predict the functional fate of proteins and identify novel bioactive peptides [20,21]. Therefore, the present study aims to systematically evaluate the hypoglycemic potential of MEP5 using an integrated *in silico* framework. The structural modeling and characterization of MEP5 were first performed. Subsequently, the direct interaction between the intact MEP5 protein and key carbohydrate-hydrolyzing enzymes were investigated. Furthermore, simulated gastrointestinal digestion was employed to identify novel MEP5-derived peptides and assess their inhibitory potential against DPP-IV via molecular docking and safety profiling. This study not only extends our understanding of the multi-target metabolic benefits of MEP5 but also provides a theoretical foundation for its application as a functional food ingredient for blood glucose management.

## 2. Materials and Methods

### 2.1. Sequence Retrieval and Physicochemical Characterization

The primary amino acid sequence of the novel fucose-specific lectin (MEP5) from *M. esculenta* was obtained from our previously reported study [17]. The physicochemical properties, including molecular weight (MW), theoretical isoelectric point (pI), instability index, aliphatic index, and grand average of hydropathicity (GRAVY), were calculated using the ExPASy 3.0 ProtParam tool (https://web.expasy.org/protparam/ (accessed on 23 March 2026)) [22]. The solubility and hydrophobicity profile of MEP5 were further analyzed using the ProtScale server (https://web.expasy.org/protscale/ (accessed on 23 March 2026)) based on the Kyte & Doolittle scale.

### 2.2. Secondary and Tertiary Structure Prediction

The secondary structural elements (*α*-helix, *β*-turn, extended strand/*β*-sheet, and random coil) of MEP5 were predicted using the SOPMA server (https://npsa.lyon.inserm.fr/cgi-bin/npsa_automat.pl?page=/NPSA/npsa_sopma.html (accessed on 23 March 2026)). To obtain the 3D conformation, the tertiary structure of MEP5 was modeled using AlphaFold3 (https://alphafoldserver.com/ (accessed on 23 March 2026)), which provides high-confidence protein structure predictions. The resulting model was validated using the SAVES v6.1 server (https://saves.mbi.ucla.edu/ (accessed on 23 March 2026)), specifically focusing on the Ramachandran plot (PROCHECK), ERRAT, and Verify 3D to evaluate the stereochemical quality of the protein backbone [23].

### 2.3. In Silico Gastrointestinal Digestion

To simulate the metabolic fate of MEP5 in the human digestive tract, *in silico* proteolysis was conducted using the Enzyme Action tool in the BIOPEP-UWM database (http://www.uwm.edu.pl/biochemia/index.php/en/biopep (accessed on 23 March 2026)). A combination of pepsin (EC 3.4.23.1), trypsin (EC 3.4.21.4), and chymotrypsin (A) (EC 3.4.21.1) was selected to mimic gastric and intestinal phases. Only peptides with a length of 2~10 amino acids were collected for subsequent bioactivity screening, as short peptides within this size range were generally recognized for their enhanced bioavailability and efficient transepithelial transport via carriers such as PepT1 [24].

### 2.4. Screening for Bioactive Hypoglycemic Peptides

The released peptides were screened for potential biological activities using the following bioinformatics tools. The potential inhibitory activity against Dipeptidyl Peptidase-IV (DPP-IV) was predicted using the StackDPPIV server (https://pmlabstack.pythonanywhere.com/StackDPPIV (accessed on 25 March 2026)) [25]. The safety profile was evaluated usingthe pepADMET server (https://pepadmet.ddai.tech/calcpep/tox/ (accessed on 25 March 2026)) [26], ToxinPred3.0 (https://webs.iiitd.edu.in/raghava/toxinpred/index.html (accessed on 25 March 2026)) [27] and AllerTOP v.2 (http://www.ddg-pharmfac.net/AllerTOP (accessed on 25 March 2026)) [28] to ensure the identified peptides were non-toxic and non-allergenic, meeting the requirements for functional food ingredients. Water solubility was estimated via the Peptide Property Calculator (https://pepcalc.com/ppc.php (accessed on 25 March 2026)). Consequently, only non-toxic and non-allergenic peptides were shortlisted for subsequent molecular docking.

### 2.5. Molecular Docking Simulation

To elucidate the molecular mechanism of the hypoglycemic effect, two types of docking simulations were performed. The interaction between the intact MEP5 protein and *α*-glucosidase (PDB ID: 3W37) was simulated using the HDOCK server (http://hdock.phys.hust.edu.cn/ (accessed on 26 March 2026)) [29] to investigate the direct enzyme inhibitory potential of the lectin.

The top-ranked hypoglycemic peptides were docked into the active site of DPP-IV (PDB ID: 6B1E). The 3D structures of the peptides were prepared using PyMOL v3.0, and docking was performed using HPEPDOCK 2.0 (http://huanglab.phys.hust.edu.cn/hpepdock/ (accessed on 2 March 2026)) [30]. The docking results were visualized, and intermolecular interactions, including binding energies, hydrogen bonds, and hydrophobic interactions, were analyzed using PyMOL v3.0 and Molecular Operating Environment 2024.06 software to identify the key amino acid residues involved in target recognition.

## 3. Results and Discussion

### 3.1. Physicochemical Profiling and Sequence Characteristics of MEP5

The physicochemical properties of a protein dictate its behavior during food processing and gastrointestinal digestion. Based on the *in silico* analysis via the ExPASy 3.0 ProtParam tool, the novel fucose-specific lectin MEP5 consists of 303 amino acids with a computed molecular weight of 33.12 kDa (Table 1), which aligns perfectly with our previous experimental SDS-PAGE isolation [17]. The computed instability index of MEP5 was 33.80. According to the criterion established by Guruprasad et al. [31], a value below 40 is predicted to be indicative of a stable protein, suggesting that MEP5 may resist spontaneous degradation during food processing and prolonged storage. However, experimental validation is required to confirm this predicted stability under actual food-manufacturing conditions. Furthermore, MEP5 exhibits a theoretical isoelectric point (pI) of 5.92 and an aliphatic index of 66.63. These parameters suggest that while protein aggregation might be avoided by steering clear of pH 5.9 during beverage processing, MEP5 is predicted to possess moderate thermal tolerance and may maintain excellent solubility at the physiological pH of the human intestine (pH ~7.4), if experimentally confirmed, this property could favor bioaccessibility for metabolic regulation [32].

**Figure 1:**
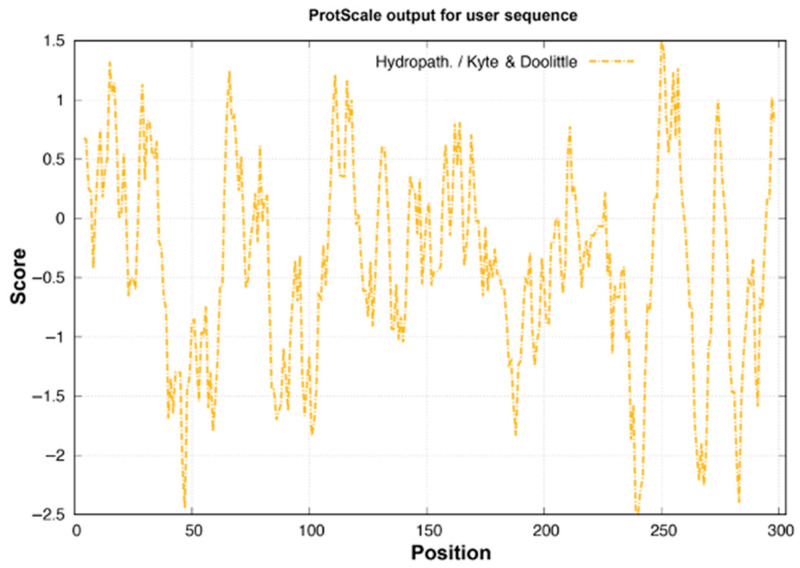
Hydropathy profile of the MEP5 sequence computed by ProtScale based on the Kyte & Doolittle scale. Regions below the zero line indicate hydrophilicity, while regions above indicate hydrophobicity.

The solubility and molecular interaction capabilities of MEP5 are further elucidated by its hydropathicity profile. The grand average of hydropathicity (GRAVY) was determined to be −0.345, reflecting a globally hydrophilic nature. As visualized in the Kyte and Doolittle hydropathy plot (Figure 1), the majority of the sequence lies below the zero baseline, with the highest hydrophilicity observed at position 240 (score: −2.500). However, this hydrophilic exterior is interspersed with distinct hydrophobic patches, notably peaking at position 250 (score: 1.500). This amphiphilic distribution may have practical implications for functional food formulation. The predominantly hydrophilic character suggests adequate water solubility, which would be favorable for incorporation into aqueous-based dietary products [33]. Concurrently, the interspersed hydrophobic patches are hypothesized to contribute to the stability of the protein core and could potentially facilitate interactions with non-polar binding pockets of hypoglycemic targets such as *α*-glucosidase and DPP-IV [34]. It must be emphasized that the connection between hydrophobic patches and enzyme binding remains speculative and requires experimental confirmation.

### 3.2. Structural Architecture and Functional Domain Analysis of MEP5

**Figure 2:**
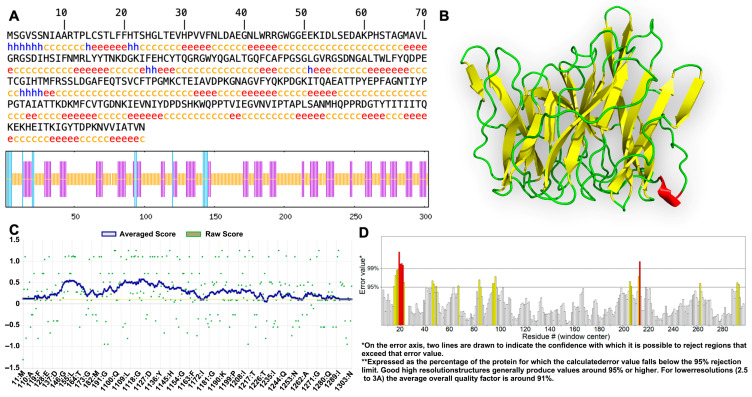
Structural characterization and comprehensive quality validation of the MEP5 model. (**A**) Secondary structure distribution mapped by the SOPMA server (Blue: *α*-helix; Red: extended strand/*β*-sheet; Orange: random coil; Green: *β*-turn). (**B**) 3D ribbon structure of MEP5 generated by AlphaFold3, highlighting the *β*-sheet-rich scaffold (yellow). (**C**) VERIFY3D plot evaluating the compatibility of the 3D model with its 1D sequence (98.68% of residues scored ≥ 0.1). (**D**) ERRAT plot showing an overall quality factor of 91.349, indicating highly reliable non-bonded atom interactions.

The spatial conformation of MEP5 is fundamental to its biological activity. Secondary structure prediction via the SOPMA server (Figure 2A) revealed a composition dominated by random coils (57.43%) and extended strand/*β*-sheet (34.65%), with minimal *α*-helices (5.28%) and *β*-turn (3.96%), which perfectly corroborates our previously reported circular dichroism experimental data [17]. To obtain an atomic-level perspective, a 3D model was constructed using AlphaFold3 (Figure 2B), displaying a dense, *β*-sheet-rich topology typical of fungal lectins. The structural reliability of this model was rigorously confirmed through a triple-validation approach: PROCHECK analysis demonstrated an exceptional backbone stereochemistry with 0.0% of residues in disallowed regions; VERIFY3D confirmed sequence-to-structure compatibility with 98.68% of residues scoring ≥0.1 (Figure 2C); and ERRAT yielded a high overall quality factor of 91.349 (Figure 2D). These robust metrics indicate that the MEP5 3D model is highly accurate and suitable for downstream molecular docking simulations.

Functionally, the validated 3D architecture offers a structural framework for understanding MEP5’s lectin activity. Our prior multi-omics profiling identified a highly conserved carbohydrate-recognition domain (CRD) located between residues 80 and 156 [17]. In the 3D model, this sequence folds into a distinct fucose-binding pocket framed by clustered *β*-strands and highly flexible loops. Given that key carbohydrate-hydrolyzing enzymes, such as *α*-glucosidase, are highly glycosylated cell-surface proteins [35], this well-defined fucose-binding cleft provides a structural rationale for its potential hypoglycemic potential. It is hypothesized that the intact MEP5 macromolecule could directly interact with the sugar moieties on these digestive enzymes, potentially creating steric hindrance that may obstruct substrate access and thereby contribute to the inhibition of their catalytic activity.

It is important to emphasize that, although MEP5 is a relatively large protein (33.12 kDa, 303 amino acids) rather than a small peptide, its bioactivity is proposed to arise from a dual-stage mechanism. Pre-digestion, the intact MEP5 macromolecule acts as a “macromolecular steric blocker” by anchoring to the glycosylated surface of *α*-glucosidase via its fucose-specific CRD, physically sealing the catalytic pocket independent of peptide size. Post-digestion, as demonstrated in the subsequent sections, the 303-amino-acid sequence serves as a reservoir of encrypted bioactive peptides. Upon gastrointestinal proteolysis, MEP5 efficiently releases numerous short oligopeptides that exhibit high solubility and potent DPP-IV inhibitory activity. This dual functionality, macromolecular enzyme blockade combined with the release of bioavailable inhibitory peptides, rationalizes the high hypoglycemic potential of MEP5 despite its larger molecular size.

### 3.3. Direct Inhibitory Potential of Intact MEP5 Against α-Glucosidase

Before exploring the digestive fate of MEP5, an investigation was conducted to determine whether the intact lectin macromolecule could exert a direct hypoglycemic effect in the upper gastrointestinal tract prior to proteolysis. To this end, protein–protein docking between the MEP5 model (ligand) and human *α*-glucosidase (PDB ID: 3W37, receptor) was performed. The input MEP5 model was pre-evaluated by ProQ, yielding an LGscore of 4.208 (indicating a good-quality model), ensuring the reliability of the docking input (Table S1) [29,36]. The docking simulation generated a highly favorable thermodynamic complex with a docking score of −281.51, indicative of a stable binding pose under the computational conditions employed. Furthermore, the empirical confidence score reached 0.9328 (well above the 0.7 threshold), supporting the plausibility that the binding between intact MEP5 and *α*-glucosidase is energetically favorable *in silico*. It should be noted, however, that docking scores and confidence metrics cannot be directly translated into quantitative inhibition constants or *in vitro* enzymatic activity, and thus, these results should be interpreted as hypothesis-generating rather than definitive.

**Figure 3:**
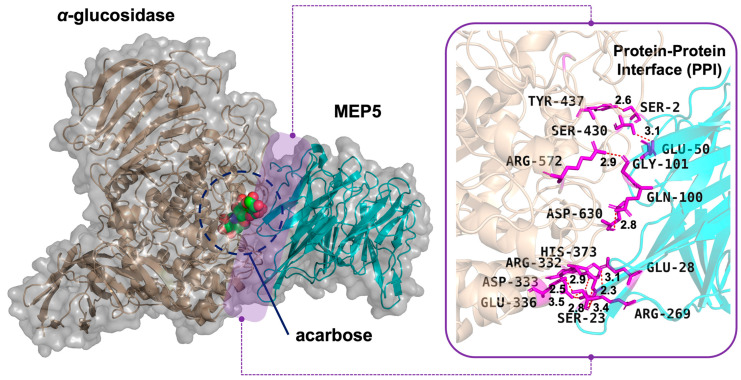
Protein–protein docking simulation revealing the direct inhibitory mechanism of intact MEP5 against *α*-glucosidase (PDB: 3W37).

The visual analysis of the docked complex (Figure 3) provides a striking mechanistic explanation for its inhibitory potential. As illustrated, the intact MEP5 macromolecule (cyan) binds tightly to the surface cleft of *α*-glucosidase (tan). As a visual reference, MEP5 positions itself directly over the catalytic pocket, overlapping with the binding region of the well-known clinical inhibitor acarbose (depicted as spheres). While this spatial coincidence is visually suggestive, it does not constitute quantitative evidence of comparable inhibitory potency. Nevertheless, this spatial arrangement suggests that MEP5 could create macromolecular steric hindrance, potentially acting as a physical barrier that may obstruct substrate access to the active site. Consequently, large carbohydrate substrates (such as starch or oligosaccharides) would be physically blocked from entering the catalytic center, thereby preventing their breakdown into absorbable glucose.

This robust steric blockade is driven by a massive network of intermolecular interactions. Analysis of the interface residues revealed that MEP5 engages with numerous critical amino acids on the surface and near the active site of *α*-glucosidase (e.g., Arg269, Asp333, Glu336, Ser430, Tyr437 and Arg572). Notably, *α*-glucosidase is a highly glycosylated enzyme. The interface data explicitly showed that specific residues of MEP5 (such as Ser2, Ser23, Glu28, Glu50, and Gln100) establish close contacts (distances < 4.9 Å) with the carbohydrate moieties of the receptor, specifically fucose (FUC 2C) and glucose/N-acetylglucosamine (GLC 1D/2D) chains. This perfectly aligns with the biological identity of MEP5 as a fucose-specific lectin [17]. These observations suggest that MEP5 may utilize its unique carbohydrate-recognition property to specifically anchor onto the glycans surrounding the *α*-glucosidase active site. This dual-action binding, protein–protein and lectin–glycan interactions would constitute a plausible first line of the hypoglycemic mechanism of MEP5 prior to its gastrointestinal digestion.

Although *α*-amylase is another well-established therapeutic target for postprandial glycemic control, the present protein–protein docking analysis focused exclusively on *α*-glucosidase. This focus was guided by the structural rationale that *α*-glucosidase is extensively glycosylated on its luminal surface, thereby presenting abundant fucose-containing glycans that could serve as specific recognition sites for the fucose-specific lectin MEP5. In contrast, the glycosylation pattern of human pancreatic *α*-amylase is comparatively limited, and its potential interaction with MEP5 remains to be clarified. Future studies incorporating both computational and experimental approaches will be required to fully elucidate whether MEP5 can also interact with *α*-amylase and contribute to the inhibition of polysaccharide digestion.

### 3.4. In Silico Gastrointestinal Digestion and Release Profile of MEP5 Peptides

Following its direct macromolecular action in the upper digestive tract, the metabolic fate of MEP5 in the stomach and intestines was simulated using the BIOPEP-UWM database with a combination of pepsin, trypsin, and chymotrypsin. The simulated proteolysis generated a comprehensive hydrolysate profile comprising 66 short peptide fragments with lengths ranging from 2 to 10 amino acids (Table S2). Statistical analysis of the cleavage patterns revealed a high frequency of low-molecular-weight fragments, predominantly composed of tetrapeptides (17 fragments), dipeptides (16 fragments), and tripeptides (15 fragments). In the context of functional nutrition, dietary proteins that can be efficiently degraded into short oligopeptides (especially 2~4 amino acids) possess tremendous value as bioactive peptide precursors. These low-molecular-weight peptides are less susceptible to further destructive luminal hydrolysis and can be efficiently transported across the intestinal epithelium into the bloodstream via specific peptide transporters (e.g., PepT1), thereby ensuring high systemic bioavailability for exerting metabolic regulation [37,38]. *In silico* proteolysis offers only a simplified model of human digestion, as it cannot incorporate dynamic factors such as pH shifts, variable enzyme activities, or food matrix effects. Accordingly, the identified peptide profile reflects a theoretical estimate under idealized conditions. Despite these limitations, this approach provides a valuable preliminary screen for identifying candidate bioactive sequences warranting further experimental study.

To identify robust hypoglycemic candidates suitable for functional food development, the 66 generated fragments were subjected to a rigorous multiparametric screening funnel. Ideal bioactive food peptides must not only exhibit strong target affinity but also guarantee strict food safety and physicochemical applicability [39]. After applying a series of computational filters, excluding sequences with predicted toxicity, predicted allergenicity, or computationally estimated poor water solubility, and retaining only those with a StackDPPIV probability > 0.5, 11 oligopeptides (3~10 amino acids) were prioritized as promising candidates (Table 2). Among those, short peptides such as IDL (predicted probability: 0.88), SAN (predicted probability: 0.83), and TDPK (predicted probability: 0.77) emerged as top-tier candidates. These soluble and completely safe fragments are predicted to effectively inhibit DPP-IV, an enzyme responsible for incretin degradation [40]. This finding powerfully suggests that, upon ingestion, MEP5 operates via a remarkable dual-stage mechanism: the intact lectin initially is predicted to obstruct carbohydrate digestion (*α*-glucosidase), while its subsequent digestion products (bioactive peptides) promote insulin secretion by targeting DPP-IV, collectively regulating glucose homeostasis.

### 3.5. Elucidating the Inhibitory Mechanism of Novel Peptides Against DPP-IV via Molecular Docking

Following the *in silico* digestion, the targeted hypoglycemic mechanism of the released bioactive peptides was elucidated through peptide–protein docking with human DPP-IV (PDB ID: 6B1E). The docking scores, which provide an estimate of the thermodynamic stability and binding affinity of the complexes, indicated that all 11 screened peptides could successfully enter the active site cavity. Notably, the top three sequences, QPPR (−154.607), DGTY (−147.913), and DPDSH (−136.708) (Table 2), exhibited favorable docking scores, suggesting the formation of highly stable, spontaneous binding conformations *in silico*. It is well established that DPP-IV is a specialized serine exopeptidase that preferentially cleaves dipeptides containing Proline (Pro) or Alanine (Ala) at the penultimate position from the N-terminus [34]. Notably, highly ranked peptides such as QPPR, DPDSH, and PDGK all possess Proline residues. This structural characteristic suggests these short peptides may mimic the natural substrates (e.g., GLP-1) and thus could potentially act as potent competitive inhibitors.

**Figure 4:**
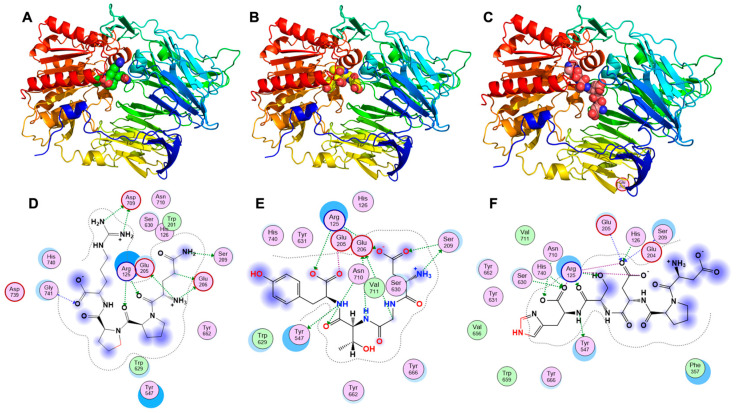
Molecular docking visualization illustrating the competitive inhibitory mechanisms of the top three MEP5-derived peptides against human DPP-IV (PDB ID: 6B1E). 3D spatial conformations showing the peptides QPPR (**A**), DGTY (**B**), and DPDSH (**C**) stably docked into the active binding cavity of the enzyme. 2D ligand–receptor interaction diagrams generated by MOE software corresponding to QPPR (**D**), DGTY (**E**), and DPDSH (**F**). The dashed lines highlight critical hydrogen bonds and strong multi-dentate ionic interactions with vital residues in the S2 pocket and the catalytic triad.

To elucidate the molecular interactions at the atomic level, the binding poses and interacting residues of the top three peptides were further analyzed using Molecular Operating Environment (MOE) software (Figure 4 and Table S3). The active site of DPP-IV features a highly polar S2 pocket (involving Arg125, Glu205, and Glu206) and a classic catalytic triad (Ser630, Asp708/709, His740) [23]. As shown in Figure 4A,D, the top-ranked peptide QPPR fits snugly into the binding cleft. Its N-terminus acts as a powerful anchor in the S2 pocket by forming an exceptionally strong hydrogen bond with Glu205 (binding energy: −13.5 kcal/mol) and multiple interactions with Glu206. Simultaneously, its C-terminal arginine establishes a dense network of multi-dentate ionic bonds (salt bridges) and hydrogen bonds with Asp709 (energies up to −6.8 kcal/mol), effectively locking the peptide adjacent to the catalytic core. Similarly, the tetrapeptide DGTY (Figure 4B,E) heavily exploits the S2 pocket. It generates a massive array of strong ionic interactions specifically with Arg125 (with energies ranging from −1.9 to −5.8 kcal/mol), while its backbone is further stabilized by hydrogen bonds with Ser209, Glu206, and Tyr547. Most strikingly, the peptide DPDSH (Figure 4C,F) suggests a direct catalytic-blocking mechanism. In addition to securing itself via Arg125 and Glu205, the backbone and side chains of DPDSH extend deep into the catalytic center, forming critical hydrogen bonds with both Ser630 (the indispensable catalytic nucleophile) and His740.

The docking results suggest that by occupying the vital S2 sub-site and directly hydrogen-bonding with the crucial residues of the catalytic triad (Ser630/His740/Asp709), these MEP5-derived peptides could create a robust steric and electrostatic barricade. This would prevent the incretin hormones (like GLP-1 and GIP) from accessing the active site. Collectively, these molecular insights provide a structural rationale for the second stage of MEP5’s proposed hypoglycemic mechanism: upon gastrointestinal digestion, the released bioactive fragments synergistically prolong the half-life of insulin-stimulating hormones by neutralizing DPP-IV activity.

Beyond the mechanistic insights provided by the present *in silico* analysis, several practical considerations are relevant to the potential application of MEP5 in functional foods. As Table 1 shows, the predicted stability of MEP5 (instability index 33.80) and its moderate aliphatic index (66.63) suggest that the protein is likely to withstand mild thermal processing, although systematic studies on its stability under various food-manufacturing conditions are warranted. With respect to bioavailability, the simulated gastrointestinal digestion demonstrated that MEP5 is efficiently degraded into short oligopeptides, which are generally favored for transepithelial transport via the PepT1 peptide transporter [33,34]. The amphiphilic character of MEP5 (GRAVY −0.345) may also influence its solubility and interaction with other food components, such as dietary fibers or lipids, which could modulate its release kinetics and bioaccessibility. Finally, while the current data do not permit a precise estimation of an effective oral dose, future *in vivo* dose–response studies will be essential to define the amount of MEP5 required to achieve a meaningful hypoglycemic effect in a dietary context. These translational aspects represent important directions for follow-up investigations.

## 4. Conclusions

This study provides a comprehensive *in silico* characterization of MEP5, a novel fucose-specific lectin from *M. esculenta*, and delineates its potential hypoglycemic mechanisms through an integrated computational framework. The intact MEP5 protein (33.12 kDa) adopts a stable, *β*-sheet-dominated tertiary structure and contains a well-defined carbohydrate-recognition domain. Protein–protein docking revealed that intact MEP5 binds directly to the surface glycans of human *α*-glucosidase, creating substantial steric hindrance that is predicted to obstruct the catalytic pocket and inhibit carbohydrate digestion. Following simulated gastrointestinal digestion, a rigorous multiparametric screening identified 11 non-toxic, non-allergenic, and water-soluble oligopeptides with potent DPP-IV inhibitory activity. Molecular docking demonstrated that the top-ranked peptides, particularly QPPR, DGTY, and DPDSH, act as competitive inhibitors by anchoring into the S2 pocket and forming direct hydrogen bonds with the catalytic triad (Ser630/His740) of DPP-IV. Collectively, these findings unravel a remarkable dual-stage hypoglycemic mechanism of MEP5: pre-digestion, the intact lectin functions as a macromolecular steric blocker targeting *α*-glucosidase via its fucose-specific carbohydrate-recognition domain; post-digestion, the protein serves as a precursor for a suite of bioavailable DPP-IV inhibitory peptides that act as incretin regulators. This dual functionality, macromolecular blockade coupled with encrypted peptide release, provides a compelling rationale for the high bioactivity of MEP5, irrespective of its relatively large molecular size.

It should be noted that this study is entirely computational; therefore, the predicted mechanisms require experimental validation. *In vitro* enzyme inhibition assays using recombinant *α*-glucosidase and DPP-IV are planned to determine IC_50_ values of intact MEP5 and its digest. Food matrix effects will also be examined under simulated meal conditions. Subsequent *in vivo* studies in diabetic rodent models will assess postprandial glycemic responses and incretin levels following oral MEP5 administration. These investigations will be essential to confirm the translational potential of MEP5 as a functional food ingredient for glucose homeostasis.

## Figures and Tables

**Table 1:** Primary amino acid sequence and key physicochemical properties of the novel fucose-specific lectin MEP5.

Protein Sequence		
MSGVSSNIAARTPLCSTLFFHTSHGLTEVHPVVFNLDAEGNLWRRGWGGEEKIDLSEDAKPHSTAGMAVLGRGSDI HSIFNMRLYYTNKDGKIFEHCYTQGRGWYQGALTGQFCAFPGSGLGVRGSDNGALTWLFYQDPETCGIHTMFRSSL DGAFEQTSVCFTPGMKCTEIAVDPKGNAGVFYQKPDGKITQAEATTPYEPFAGNTIYPPGTAIATTKDKMFCVTGD NKIEVNIYDPDSHKWQPPTVIEGVNVIPTAPLSANMHQPPRDGTYTITIITQKEKHEITKIGYTDPKNVVIATVN		
Formula	C_1474_H_2251_N_395_O_448_S_14_	
Number of amino acids	Theoretical pI	Molecular weight
303	5.92	33,122.31
Instability index	Aliphatic index	Grand average of hydropathicity (GRAVY)
33.80	66.63	−0.345

**Table 2:** Predicted physicochemical properties, safety profiles, and predicted DPP-IV inhibitory probabilities of the top 11 screened bioactive peptides derived from the simulated gastrointestinal digestion of MEP5.

No.	1	2	3	4	5	6	7	8	9	10	11
Sequence	IDL	SAN	TDPK	EITK	QPPR	DGTY	PDGK	DPDSH	TEVH	DGK	GVR
Number of amino acids	3	3	4	4	4	4	4	5	4	3	3
Probable allergen	NO	NO	NO	NO	NO	NO	NO	NO	NO	NO	NO
Water solubility	Good	Good	Good	Good	Good	Good	Good	Good	Good	Good	Good
Toxin	NO	NO	NO	NO	NO	NO	NO	NO	NO	NO	NO
Probability (StackDPPIV)	0.88	0.83	0.77	0.75	0.63	0.63	0.62	0.59	0.58	0.56	0.53
Hydrophobicity	0.18	−0.22	−0.52	−0.29	−0.65	−0.18	−0.43	−0.43	−0.17	−0.55	−0.35
Steric hindrance	0.66	0.6	0.58	0.65	0.52	0.67	0.62	0.48	0.48	0.71	0.69
Amphipathicity	0	0	0.92	1.23	0.93	0	0.92	0.29	0.68	1.22	0.82
pI	3.8	5.88	6.19	6.35	10.11	3.8	6.19	4.2	5.25	6.19	10.11
Mol w.t. (Da)	359.46	290.3	459.54	489.62	496.61	454.48	415.49	569.58	484.56	318.36	330.42
Docking Score	−96.748	−87.962	−108.956	−105.661	−154.607	−147.913	−122.504	−136.708	−134.968	−75.181	−126.336

## Data Availability

The original contributions presented in this study are included in the article/Supplementary Materials. Further inquiries can be directed to the corresponding authors.

## Supplementary Materials

The following supporting information can be downloaded at: https://www.mdpi.com/article/10.3390/foods15091493/s1, Table S1. Quality report of MEP5 protein by ProQ v1.2. Table S2. The simulated proteolysis generated a comprehensive hydrolysate profile comprising 66 short peptide fragments and their safety profiles and predicted DPP-IV inhibitory probabilities. Table S3. The interactive residues of the top three peptides analyzed using Molecular Operating Environment (MOE) software.
