# Supplementary material for: In Silico Structural Characterization and Hypoglycemic Potential of a Novel Fucose-Specific Lectin (MEP5) from Morchella esculenta
Source: Foods. 2026 Apr 24;15(9):1493. doi: 10.3390/foods15091493 (PMC13164499; doi:10.3390/foods15091493)
Supplement: Supplementary file 1 [file foods-15-01493-s001.zip › foods-4261684-supplementary.pdf]

In Silico Structural Characterization and Hypoglycemic Potential of a Novel Fucose-Specific Lectin (MEP5) from *Morchella esculenta*

Wanchao Chen, Peng Liu, Wen Li, Di Wu, Zhong Zhang, Yan Yang*

Institute of Edible Fungi, Shanghai Academy of Agricultural Sciences; National Engineering Research Center of Edible Fungi; Key Laboratory of Edible Fungi Resources and Utilization (South), Ministry of Agriculture and Rural Affairs, P.R. China

Table S1 Quality report of MEP5 protein by ProQ v1.2

Index	Results	
LGscore	4.208	
MaxSub	0.366	
Different ranges of protein model quality by LGscore or MaxSub		
Correct	Good	Very good
1.5 < LGscore	3.0 ≤ LGscore < 5.0	5.0 ≤ LGscore
0.1 < MaxSub	0.5 ≤ MaxSub < 0.8	0.8 ≤ MaxSub

Table S2 The simulated proteolysis generated a comprehensive hydrolysate profile comprising 66 short peptide fragments and their safety profiles and predicted DPP-IV inhibitory probabilities

NO.	Peptides	Length	Toxicity	ALLERGEN	Water solubility	Probability (StackDPPIV sever)
1	SGVSSN	6	NO	NO	Poor	0.73
2	IAAR	4	NO	YES	Good	0.47
3	TPL	3	NO	YES	Poor	0.73
4	CSTL	4	NO	NO	Poor	0.75
5	TSH	3	NO	YES	Poor	0.81
6	GL	2	NO	NO	Poor	1.00
7	TEVH	4	NO	NO	Good	0.58
8	PVVF	4	NO	YES	Poor	0.91
9	DAEGN	5	NO	YES	Good	0.42
10	GW	2	NO	YES	Poor	1.00
11	GGEEK	5	NO	YES	Good	0.51
12	IDL	3	NO	NO	Good	0.88
13	SEDAK	5	NO	NO	Good	0.39
14	PH	2	NO	NO	Poor	1.00
15	STAGM	5	NO	YES	Poor	0.47
16	AVL	3	NO	NO	Poor	1.00
17	GR	2	NO	NO	Good	0.95
18	GSDIH	5	NO	YES	Good	0.26
19	SIF	3	NO	NO	Poor	0.98
20	TN	2	NO	NO	Good	1.00
21	DGK	3	NO	NO	Good	0.56
22	IF	2	NO	NO	Poor	1.00
23	EH	2	NO	NO	Good	1.00
24	CY	2	NO	NO	Poor	0.61
25	TQGR	4	NO	NO	Good	0.41
26	GW	2	NO	YES	Poor	1.00
27	QGAL	4	NO	YES	Poor	0.92
28	TGQF	4	YES	YES	Poor	0.50
29	CAF	3	NO	YES	Poor	0.63
30	PGSGL	5	NO	NO	Poor	0.87
31	GVR	3	NO	NO	Good	0.53

32	GSDN	4	NO	YES	Good	0.75
33	GAL	3	NO	NO	Poor	1.00
34	TW	2	NO	YES	Poor	1.00
35	QDPETCGIH	9	NO	YES	Good	0.64
36	TM	2	NO	NO	Poor	1.00
37	SSL	3	NO	YES	Poor	0.96
38	DGAF	4	YES	YES	Good	0.76
39	EQTSVCF	7	NO	YES	Poor	0.45
40	TPGM	4	NO	NO	Poor	0.60
41	CTEIAVDPK	9	NO	YES	Good	0.62
42	GN	2	NO	NO	Good	1.00
43	AGVF	4	YES	NO	Poor	0.63
44	QK	2	NO	NO	Good	0.97
45	PDGK	4	NO	NO	Good	0.62
46	ITQAEATTPY	10	NO	YES	Poor	0.48
47	EPF	3	NO	YES	Good	0.95
48	AGN	3	YES	YES	Poor	0.96
49	TIY	3	NO	NO	Poor	0.90
50	PPGTAIATTK	10	NO	NO	Poor	0.32
51	DK	2	NO	NO	Good	0.99
52	CVTGDN	6	NO	YES	Good	0.39
53	IEVN	4	NO	YES	Good	0.86
54	IY	2	NO	YES	Poor	0.94
55	DPDSH	5	NO	NO	Good	0.59
56	QPPTVIEGVN	10	NO	YES	Poor	0.86
57	VIPTAPL	7	NO	YES	Poor	0.78
58	SAN	3	NO	NO	Good	0.83
59	QPPR	4	NO	NO	Good	0.63
60	DGTY	4	NO	NO	Good	0.63
61	TITIITQK	8	NO	YES	Poor	0.70
62	EK	2	NO	NO	Good	0.97
63	EITK	4	NO	NO	Good	0.75
64	IGY	3	YES	NO	Poor	0.92
65	TDPK	4	NO	NO	Good	0.77
66	VVIATVN	7	NO	YES	Poor	0.58

Table S3 The interactive residues of the top three peptides analyzed using Molecular Operating Environment (MOE) software

Ligand: QPPR		Receptor: 6B1E			Interaction	Distance	E(kcal/mol)
N	1	OE2	GLU	205	H-donor	2.63	-13.5
N	1	OE1	GLU	206	H-donor	3.72	-5.0
N	1	OE2	GLU	206	H-donor	3.50	-4.2
CG	6	OE1	GLU	206	H-donor	3.40	-0.9
NE2	8	OG	SER	209	H-donor	3.51	-1.2
NH1	33	OD1	ASP	709	H-donor	2.86	-3.5
NH1	33	OD2	ASP	709	H-donor	2.80	-4.3
NH2	34	OD2	ASP	709	H-donor	2.70	-2.9
O	4	NH2	ARG	125	H-acceptor	3.19	-1.1
O	13	NH1	ARG	125	H-acceptor	3.15	-5.0
O	13	NH2	ARG	125	H-acceptor	3.19	-2.3
O	27	CA	GLY	741	H-acceptor	2.92	-0.8
N	1	OE2	GLU	205	Ionic	2.63	-7.5
N	1	OE1	GLU	206	Ionic	3.72	-1.2
N	1	OE2	GLU	206	Ionic	3.50	-1.9
NH1	33	OD1	ASP	709	Ionic	2.86	-5.5
NH1	33	OD2	ASP	709	Ionic	2.80	-6.0
NH2	34	OD2	ASP	709	Ionic	2.70	-6.8
**Ligand: DGTY**		**Receptor: 6B1E**			**Interaction**	**Distance**	**E(kcal/mol)**
N	1	OG	SER	209	H-donor	3.57	-4.3
CA	10	OE1	GLU	206	H-donor	2.76	-1.7
N	13	OE2	GLU	206	H-donor	3.68	-1.1
N	20	OH	TYR	547	H-donor	3.11	-1.1
OD1	7	OG	SER	209	H-acceptor	3.59	-0.6
OD2	8	CD	ARG	125	H-acceptor	3.19	-1.1
O	16	ND2	ASN	710	H-acceptor	2.98	-1.2
OXT	32	NH1	ARG	125	H-acceptor	2.83	-4.9
OXT	32	NH2	ARG	125	H-acceptor	3.22	-0.8
OD2	8	NE	ARG	125	Ionic	3.85	-0.8
OD2	8	NH1	ARG	125	Ionic	3.88	-0.7
O	23	NH1	ARG	125	Ionic	3.49	-1.9
O	23	NH2	ARG	125	Ionic	3.39	-2.3
OXT	32	NH1	ARG	125	Ionic	2.83	-5.8
OXT	32	NH2	ARG	125	Ionic	3.22	-3.1

**Ligand: DPDSH**		**Receptor: 6B1E**			**Interaction**	**Distance**	**E(kcal/mol)**
N	30	OH	TYR	547	H-donor	2.79	-1.8
OD1	22	NE2	HIS	126	H-acceptor	2.60	-2.2
OD1	22	CA	GLU	205	H-acceptor	3.44	-1.1
OD1	22	CA	SER	209	H-acceptor	3.79	-0.5
O	27	NH2	ARG	125	H-acceptor	3.05	-0.8
OG	29	NH1	ARG	125	H-acceptor	2.89	-1.3
OG	29	NH2	ARG	125	H-acceptor	3.00	-1.3
O	33	OG	SER	630	H-acceptor	2.70	-0.5
O	33	NE2	HIS	740	H-acceptor	2.91	-0.5
OXT	40	ND2	ASN	710	H-acceptor	3.21	-0.5
OD1	22	NE	ARG	125	Ionic	3.67	-1.3
OD2	23	NE	ARG	125	Ionic	3.91	-0.7
O	33	NH2	ARG	125	Ionic	3.81	-0.9
